# Stage IV SMARCA4‐Deficient Undifferentiated Malignant Neoplasm With Primary Cutaneous Origin and Early Distant Metastases

**DOI:** 10.1155/crdm/9982655

**Published:** 2026-01-16

**Authors:** Katlyn M. Smaha, Matthew Willett, David E. Kent

**Affiliations:** ^1^ Medical College of Georgia at Augusta University, Augusta, Georgia, USA, augusta.edu; ^2^ Naval Hospital Camp Pendleton, Camp Pendleton, California, USA; ^3^ Skin Care Physicians of Georgia, Macon, Georgia, USA; ^4^ Atrium Health Navicent the Medical Center, Macon, Georgia, USA

## Abstract

SMARCA4‐deficient undifferentiated malignant neoplasms (SD‐UMNs) are a recently recognized group of malignant epithelioid tumors, associated with mutations in the SWItch/Sucrose nonfermentable chromatin remodeling complex. To our knowledge, there have been only three cases of SD‐UMNs primary to the skin. We report a rare case of primary cutaneous SD‐UMN in an 85‐year‐old male with a former 25‐pack‐year smoking history. Unlike previous cases, he notably was diagnosed as Stage IV upon presentation, with metastatic involvement of lymph nodes and liver. Our case highlights the importance of recognizing SD‐UMN from other types of poorly differentiated cutaneous epithelioid malignant neoplasms, given its aggressive nature and potential for targeted therapies. It also adds to the growing but still limited understanding of the clinical and histopathological features of this rare malignancy.

## 1. Introduction

SMARCA4‐deficient undifferentiated malignant neoplasms (SD‐UMNs) are a category of highly aggressive tumors displaying an epithelioid morphology [1]. They are defined by the absence of functional SMARCA4, detectable by immunohistochemistry [2, 3]. SMARCA4 (also known as transcriptional activator BRG1) is an ATPase and crucial component of the SWItch/Sucrose nonfermentable (SWI/SNF) chromatin remodeling complex [4]. Its loss of function is well established for oncogenesis [5, 6]. Among this tumor family, SMARCA4‐deficient thoracic sarcoma is the most well‐known and first recognized. It most often affects male smokers in their fifth and sixth decades due to a smoking‐related mutation [7].

Three cases of SD‐UMNs primary to the skin have been reported [1, 8]. Notably, none of these patients had metastatic disease at presentation. We report a rare case of primary cutaneous SD‐UMN. In contrast to previous cases, our patient had metastatic disease at presentation, demonstrating a comparable aggressiveness to other SMARCA4‐deficient visceral neoplasms.

## 2. Case Presentation

An 85‐year‐old male with a history of actinic keratoses presented for a new skin lesion on the right distal forearm enlarging over 8 months. He reported intermittent bleeding, soreness, and occasional burning. He had no history of melanoma or nonmelanoma skin cancer. He had a former 25‐pack‐year smoking history but quit tobacco use over 30 years ago.

**Figure 1:**
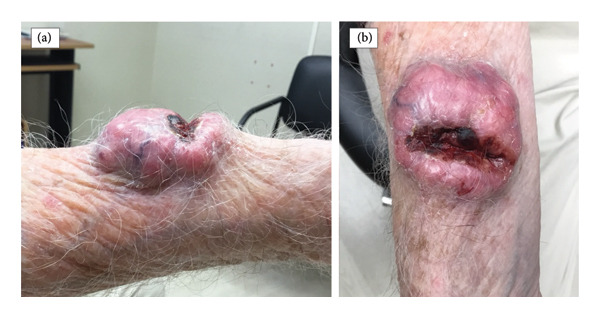
Right ventral lateral distal forearm with a 5.6 × 5 cm pink, irregular, fungating nodule with central cavitation and bleeding.

On examination, there was a 5.6 × 5 cm pink, irregular, fungating nodule with central cavitation and bleeding, located on the right ventral lateral distal forearm (Figures 1(a) and 1(b)). The patient had multiple, enlarged, mildly tender, mobile right axillary lymph nodes.

**Figure 2:**
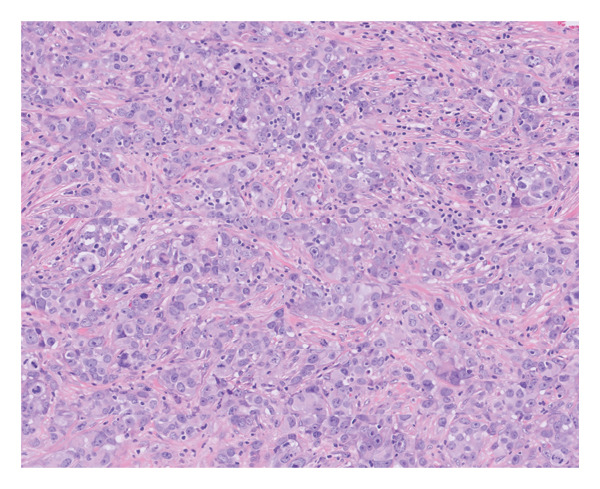
High‐power view showing poorly differentiated epithelioid malignant neoplasm consisting of cells with abundant eosinophilic cytoplasm, vesicular nuclei, and a high mitotic rate (H&E, original magnification × 400).

Shave biopsy was obtained. Results demonstrated a poorly differentiated epithelioid malignant neoplasm, consisting of cells with abundant amphophilic cytoplasm and large atypical vesicular nuclei, showing a high mitotic rate (Figure 2). A few of the cells contained rhabdoid cytoplasmic inclusions. Immunostaining showed multifocal positivity for pan‐keratin, EMA, and SOX2 along with focal positivity for CAM5.2. S100 protein, SOX10, CD34, p40, LCA, desmin, and claudin‐4 were negative. Importantly, the neoplastic cells were entirely negative for SMARCA4 (Table 1). The morphologic appearance and immunophenotype were most consistent with a SD‐UMN.

**Table 1:** Immunohistochemistry profile of primary cutaneous SMARCA4‐deficient undifferentiated malignant neoplasm.

Marker	Result	Diagnostic significance
Pan‐keratin	Multifocal positive	Confirms epithelial differentiation
Epithelial membrane antigen (EMA)	Multifocal positive	Supports epithelial origin
SOX2	Multifocal positive	Biomarker for stem cells/undifferentiated phenotype; reported in SMARCA4‐deficient tumors
CAM5.2	Focal positive	Cytokeratin marker, reinforces epithelial differentiation
S100 protein	Negative	Rules against melanocytic or neural differentiation
SOX10	Negative	Excludes melanoma and other tumors of neural crest origin (e.g., schwannomas)
CD34	Negative	Rules against vascular and some fibroblastic tumors
p40	Negative	Excludes squamous differentiation
Leukocyte common antigen (LCA), also known as CD45	Negative	Excludes hematolymphoid neoplasms
Desmin	Negative	Rules against myogenic differentiation
Claudin‐4	Negative	Excludes certain epithelial carcinomas (e.g., adenocarcinomas)
SMARCA4	Negative	Loss of SMARCA4 expression, diagnostic hallmark of SMARCA4‐deficient tumors

The patient was referred to a large cancer center. Computed tomography (CT) imaging with intravenous contrast of the chest and abdomen/pelvis revealed right axillary and right subpectoral lymphadenopathy and several small hypodensities in the liver. Right axillary lymph node biopsy was positive for metastasis. Given the absence of primary thoracic tumor, these findings were consistent with Stage IV primary cutaneous SMARCA4‐deficient undifferentiated malignant tumor.

Orthopedic oncology resected the primary soft tissue tumor given the mass was bleeding, painful, and rapidly growing. Due to positive deep margins of resection, the patient underwent subsequent debridement and intraoperative radiation. Following surgery, the patient was started on external beam hypofractionated radiation therapy at 15fx to 42.75 Gy of the primary cutaneous tumor site given the surgical pathology indicated a positive deep surgical margin. Chemotherapy was initially held given a postoperative polymicrobial infection at the surgical site. Plans to begin systemic therapy with gemcitabine and docetaxel were discussed with the patient. However, follow‐up CT showed worsening hepatic and nodal metastatic disease with possible inferior vena cava and left iliac metastases. Given aggressive metastatic disease, patient fatigue, and lack of meaningful family or social support, the patient opted against chemotherapy and continued with radiation therapy with palliative intent.

## 3. Discussion

SD‐UMNs have been described as primary tumors arising in the lungs, gastrointestinal tract, sinonasal tract, and gynecologic tract, with only three reported cases of primary cutaneous involvement to date [1, 8, 9]. All three reported primary cutaneous cases occurred in older men and demonstrated ultraviolet A (UVA)–associated mutational profiles [1, 8]. Of these cases, two patients remained recurrence‐free at 1‐year follow‐up, whereas one experienced a more aggressive disease course, developing metastatic disease despite initial treatment. Notably, none of the previously reported cutaneous cases presented with metastatic disease, in contrast to SMARCA4‐deficient visceral malignancies, which are frequently metastatic at the time of diagnosis [9]. Therefore, the true incidence, natural history, treatment response, and prognosis of primary cutaneous SD‐UMNs remain poorly defined. Our patient’s case expands upon the limited literature on primary cutaneous SD‐UMNs because he presented with metastatic involvement of both lymph nodes and liver.

The aggressiveness of SD‐UMNs may be attributed to the direct effects of SMARCA4 loss on SWI/SNF function and chromatin regulation [10]. The SWI/SNF complex is an important tumor suppressor, involved in DNA‐damage repair, transcription of genes normally repressed by chromatin, and cell cycle progression [11–13]. It has been specifically linked to the repair of UV‐induced DNA damage [14]. While the precise pathogenesis of primary cutaneous SD‐UMNs is not well understood, UVA‐related mutation profiles are reported [1]. This is consistent with their cutaneous origin and the SWI/SNF complex’s role in UV‐induced DNA damage repair.

On histopathology, SD‐UMN typically shows an undifferentiated epithelioid large cell or rhabdoid cytomorphology [15, 16]. Vesicular chromatin with amphophilic cytoplasm and variably prominent nucleoli, atypical mitotic figures, and geographic necrosis may be seen [15, 16]. The lack of distinct differentiation in appearance poses a diagnostic challenge. The differential diagnosis for poorly differentiated epithelioid malignancies in the skin is broad; it includes poorly differentiated squamous cell carcinoma, melanoma, Merkel cell carcinoma, epithelioid sarcoma, adnexal carcinomas, clear cell sarcoma, high‐grade lymphomas, and epithelioid angiosarcoma [17]. It is also important to consider cutaneous metastasis. Leckey et al. reported a case of SMARCA4‐deficient thoracic sarcoma metastasis to the skin [18]. For our patient, given the lack of primary thoracic tumor on imaging, metastasis was less likely. Metastasis from a spontaneously regressed visceral primary tumor was also not likely, as this is not well described.

Definitive diagnosis is made by immunohistochemistry demonstrating loss of SMARCA4 [2, 3]. Less specific markers include immunopositivity for pan‐keratin, SOX2, and EMA [1, 18]. In cutaneous cases, molecular analysis may be useful for diagnosis and UVA‐related mutation profiles [1]. Given the known aggressiveness of SMARCA4‐deficient tumors, initial workup with PET/CT, along with comprehensive staging, is recommended to assess for metastatic disease and guide management. While additional workup with PET or endoscopy was not performed for our patient, the combination of a solitary, enlarging cutaneous mass, regional nodal involvement, and absence of detectable primary tumors elsewhere on CT imaging strongly supported the cutaneous origin of the neoplasm.

While there are no established treatment guidelines for SD‐UMN, treatment of the primary cutaneous tumor includes surgical excision [1]. This may be combined with radiation therapy, chemotherapy, and/or immune checkpoint inhibitor therapy [1, 19]. In some cases, radiation may be used as palliative therapy [20]. Additionally, targeted therapies against Enhancer of zeste Homolog 2 (EZH2), which plays a role in the oncogenesis of SWI/SNF mutations, are in clinical trials [21].

The prognosis for most SMARCA4‐deficient tumors is poor. However, less is known about the prognosis of primary cutaneous SD‐UMN. Unlike the three previously reported cutaneous cases, our patient uniquely presented with nodal and visceral metastases at initial presentation, thereby demonstrating that primary cutaneous tumors can display aggressiveness similar to SMARCA4‐deficient visceral primaries. This finding underscores the importance of considering SMARCA4 immunohistochemistry when evaluating undifferentiated cutaneous tumors, as well as performing comprehensive systemic staging when a primary cutaneous SD‐UMN is diagnosed. As awareness of this complex malignancy grows, further research into its pathogenesis and molecular targeting will lead to improved clinical practices and therapies for its management.

## Consent

All the patients allowed personal data processing, and informed consent was obtained from all individual participants included in the study.

## Conflicts of Interest

The authors declare no conflicts of interest.

## Funding

The authors declare that there is no current or recent funding that might influence this article.

## Data Availability

The data used to support the findings of this study are included within the article.
